# Transplacental Passage and Fetal Effects of Antineoplastic Treatment during Pregnancy

**DOI:** 10.3390/cancers14133103

**Published:** 2022-06-24

**Authors:** Silvia Triarico, Serena Rivetti, Michele Antonio Capozza, Alberto Romano, Palma Maurizi, Stefano Mastrangelo, Giorgio Attinà, Antonio Ruggiero

**Affiliations:** 1 UOSD di Oncologia Pediatrica, Dipartimento di Scienze della Salute della Donna, del Bambino e di Sanità Pubblica, Fondazione Policlinico Universitario A. Gemelli IRCCS, Largo Agostino Gemelli 8, 00168 Rome, Italy; silvia.triarico@guest.policlinicogemelli.it (S.T.); serena.rivetti@gmail.com (S.R.); alberto.romano@guest.policlinicogemelli.it (A.R.); palma.maurizi@unicatt.it (P.M.); stefano.mastrangelo@unicatt.it (S.M.); giorgio.attina@policlinicogemelli.it (G.A.); 2 Scuola di Specializzazione in Pediatria, Università Cattolica del Sacro Cuore, Largo Francesco Vito 1, 00168 Rome, Italy; 3 Pediatric Oncology Unit, AORN Santobono-Pausilipon, 80123 Napoli, Italy; micheleantoniocapozza@gmail.com; 4 Dipartimento di Scienze della Vita e Sanità Pubblica, Università Cattolica del Sacro Cuore, Largo Francesco Vito 1, 00168 Rome, Italy

**Keywords:** pregnancy, transplacental passage, fetus, newborn, cancer, chemotherapy, targeted agents

## Abstract

**Simple Summary:**

In this paper we perform an introduction about pregnancy-associated cancer (PAC) and transplacental passage of antineoplastic agents. Furthermore, we describe therapeutic use and potential toxic effects of chemotherapeutic drug (alkylating agents, antimetabolites agents, anthracyclines, topoisomerase inhibitors, antimitotic agents, actinomycin-D, bleomycin) and targeted agents during pregnancy. This manuscript may be a useful and practical guide for the management of PAC, which is a challenge for clinicians that have to consider alike maternal benefits and fetal potential risks correlated to the antineoplastic treatment.

**Abstract:**

The incidence of PAC is relatively infrequent among pregnant women. However, it has gradually increased in recent years, becoming a challenging area for clinicians that should take into account in the same way maternal benefits and fetal potential risks correlated to the antineoplastic treatment. None of the antineoplastic drugs is completely risk-free during the pregnancy, the timing of exposure and transplacental transfer properties influence the toxicity of the fetus. Despite the lack of guidelines about the management of PAC, several studies have described the use and the potential fetal and neonatal adverse events of antineoplastic drugs during pregnancy. We provide a review of the available literature about the transplacental passage and fetal effects of chemotherapy and targeted agents, to guide the clinicians in the most appropriate choices for the management of PAC.

## 1. Introduction

Cancer is relatively infrequent among pregnant women, complicating about 0.1% of all pregnancies annually. Breast cancer, cervical cancer, melanoma, lymphoma, leukaemia, and ovarian cancer show a rising incidence curve during the reproductive years, accounting for 70–80% of PAC [1,2]. However, also other tumors (such as thyroid, colorectal, brain, kidney, and soft tissue sarcomas) may occur during pregnancy [3]. Metastasis to the fetus is rare, but has been reported in pregnancies complicated by melanoma, hematopoietic malignancies, and lung cancer; furthermore, the risk of transplacental metastases is about 30% for the melanoma [4].

The incidence of PAC is expected to grow in the coming years, likely because of the growing age of childbearing, associated with amplified risk of developing age-dependent malignancies. Breast cancer is the most common PAC, reported in about 0.04% of pregnancies and significantly correlated with age [5,6]. In fact, among the Italian population, its incidence seems to be four times higher for pregnant women aged 40 years than those aged 30 years [7].

It should be noted that there is no reliable evidence that pregnancy increases the risk of developing cancer or of worsening its prognosis, although its management strategy generates clinical and ethical challenges regarding fetal and maternal health. The management of PAC must take into account the same way maternal life-saving benefits and fetal potential life-threatening risks correlated to the treatment [8].

Antineoplastic drugs are often essential for the optimal treatment of cancer, but none of them is completely safe for use in pregnancy. Their toxicity depends mainly on the timing of exposure to chemotherapy and on the transplacental transfer of the drug from the mother to the fetus [9]. In the first ten days after conception embryonal cells are omnipotent and the “all-or-nothing” phenomenon is observed, after the exposure to a toxic drug the embryo survives if few cells are destroyed, while it dies when the number of destroyed cells is elevated [10]. The administration of antineoplastic drugs is contraindicated from 10 days after conception and until 14 weeks of pregnancy because of the elevated risk of major malformations (to the hearth, neural tube, upper and lower limbs, eyes, palate and ears) that may occur during the organogenesis [11]. After the first trimester, the administration of chemotherapy carries an increased risk of preterm birth, intrauterine growth restriction (IUGR), low birth weight and bone marrow suppression, but also of minor anomalies and functional defects for eyes, gonads and genitalia, hematopoietic system and central nervous system [12]. However, when the treatment cannot be postponed to childbirth, chemotherapy should be performed during the fetal phase (second and third trimester). An interval of at least three weeks should be considered between the end of chemotherapy and the delivery, to avoid it during the period of maternal myelosuppression and drug accumulation in the newborn. Delivery should not occur before 35–37 weeks, to avoid iatrogenic prematurity [13].

In this narrative review, we provide an updated report on the main antineoplastic drugs and targeted agents that may be used during pregnancy, on their transplacental passage and adverse events that may occur in the fetus and newborns.

In Table 1 we summarize the main effects on pregnancy and fetal outcomes of each antineoplastic agent, with the hope that it will be useful to help clinicians for the most appropriate choices in the management of PAC.

In Table 2 we divided antineoplastic agents relatively safe after the first trimester from those which are absolutely contraindicated.

## 2. Transplacental Passage of Chemotherapy

The placenta is the interface between the mother and the fetus which plays a crucial role in fetal development, providing nutrients and oxygen required for fetal tissue growth. Moreover, the placenta creates an immunologic barrier between the fetus and the mother and produces cytokines and hormones necessary for fetal energy metabolism [71]. The structure of the placenta varies from one species to another. The human placenta is classified as “hemocorial” type, as the maternal and fetal blood are separated by a single syncytiotrophoblast cell layer. In contrast, the placenta of mice and rats is defined as “hemotrichorial”, because of the presence of three cell layers (cytotrophoblast, syncytiotrophoblast I and syncytiotrophoblast II), that separate maternal from fetal blood [72]. The main functional units of the human placenta are the chorionic villi, formed by fetal vessels and covered by endothelium inside and the trophoblasts outside-facing maternal blood. Maternal blood penetrates the placenta through the spiral arteries that carry blood directly into the intervillous space. Maternal blood circulates in the intervillous space, providing the fetus oxygen and nutrients and discharging the waste and toxic substances (such as xenobiotics); fetal blood circulates inside the villi, through which the fetus enters the maternal metabolism [73]. The first trimester of pregnancy is crucial for an adequate maternal-placental flow. In fact, during the first-trimester trophoblast cells invade the spiral arteries, causing the replacement of the smooth and elastic muscle tissue of endothelial cells with trophoblast cells [74]. These important modifications that involve the spiral arteries produce a reduction of resistances and around the 16th–18th week of gestation, the spiral vessels are transformed into dilated vessels unresponsive to vasoconstriction, allowing a low-pressure circulation to the placenta. Alteration of this mechanism may produce high placental resistance that is typically associated with obstetric pathologies, such as preclampsia and IUGR [75].

In conditions of physiological placental development, maternal and fetal blood establish a functional relationship, but they are separated into two different circulations by the placental barrier. The placental barrier is an endothelial cell layer, consisting of a thin layer of connective tissue and a continuous syncytiotrophoblast that covers an inner mononuclear layer of cytotrophoblasts [76]. By the end of the first trimester, maternal blood begins to flow from the maternal spiral arteries into the intervillous space and the placental barrier is the interface of maternal-fetal exchange. The placental barrier and maternal-fetal diffusion distance gradually thin, proceeding with gestation, decreasing from over 50 μm in the late second month to less than 5 μm by the 37th week of pregnancy [77].

**Figure 1:**
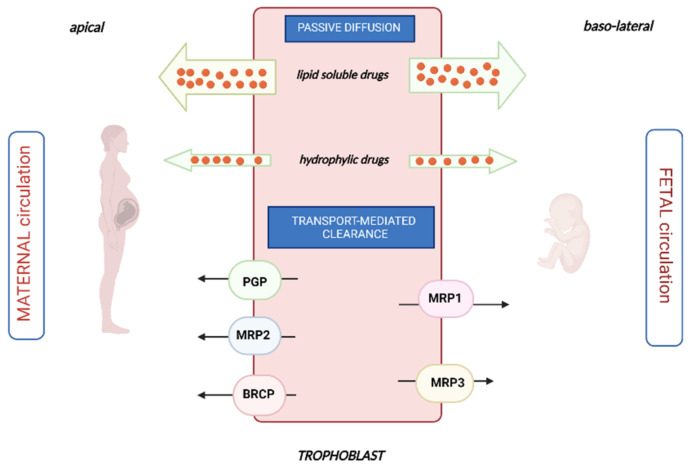
Mechanisms of transplacental passage of drugs.

All anticancer agents can theoretically cross the placental barrier, but the extent of placental transfer varies substantially from one compound to another, based on their physicochemical properties (such as molecular weight, lipophilia, ionization at physiological pH and plasma protein binding). Generally, highly lipophilic, low-molecular-weight (<500 Da) molecules are not ionized at physiological pH and are weakly bound or unbound to plasma proteins (such as carboplatin), consequently, they are likely to cross the placental barrier through passive diffusion more easily than hydrophilic ionized molecules. The amount of drug that crosses the placenta through passive diffusion depends on the concentration of the drug in the maternal circulation. Moreover, it does not require the input of energy and is not saturable nor subject to competitive inhibition [78]. Drugs with higher molecular weight that are more hydrophilic may be pumped across the placental barrier by various active transporters situated on both the syncytiotrophoblast and fetal capillary endothelium of the placental barrier, thus they pump drugs into or out of the syncytiotrophoblast. These active transporters are powered by adenosine triphosphate (ATP), they act against a concentration gradient and may be saturated. They are represented mainly by P-glycoprotein (Pgp or MDR1), multidrug resistance protein 1,2,3 (MRP1, MRP2 and MRP3) and breast cancer resistance protein (BCRP), as described in Figure 1 [79].

Pgp is located on the maternal side of the placental barrier and shows a great capacity to carry its substrates from the fetal to the maternal direction, playing a feto-protective role. Consequently, drugs that are known as substrates of Pgp (such as paclitaxel) should be preferred to others during pregnancy. MRP1 and MRP3 are particularly copious in fetal endothelial cells of the placenta microcapillary, whereas MRP2 was detected on the maternal side, in the apical membranes of the syncytiotrophoblast. MRP and BRCP proteins seem to play a feto-protective role, by the removal of metabolites from the fetus to the mother, creating pharmacological sanctuaries. BCRP is highly expressed on the maternal side and it may render tumor cells resistant to the anticancer drugs topotecan, mitoxantrone, doxorubicin and daunorubicin, reducing their passage to the fetus [80].

Van Calsteren et al. investigated the transplacental transfer of different chemotherapeutic drugs from mother to fetus in a mouse and baboon model. The baboon model should be considered an animal model with a close phylogenetic relationship to humans concerning embryological development, reproductive physiology, placental structure and function and drug metabolism. In the baboon model, they found fetal plasma concentrations differ significantly for each chemotherapeutic drug. Anthracyclines (doxorubicin and epirubicin) and taxanes (paclitaxel) showed a limited transplacental transfer (<10% and <2%, respectively). Conversely, carboplatin was the only one found in higher concentration (>55%) in the fetal compartment, as an expression of its elevated transplacental passage [81].

Finally, physiological changes in the metabolism of pregnant women may influence the pharmacokinetics of drugs, resulting in a lower plasma exposure [82]. The volume of distribution of many drugs increases by almost 50% during pregnancy, reducing their peak plasma concentration and enhancing the distribution volume for water-soluble drugs. Moreover, renal clearance is improved, due to the augmented renal blood flow and glomerular filtration rate (GFR). In addition, upregulation of liver oxidative metabolism is observed, with the improved activity of cytochrome P450 isoform 3A4, which potentially leads to reduced maternal exposure to several drugs that are metabolized by this isoenzyme. However, there is no sure evidence of lower therapeutic responses in pregnant than in non-pregnant women and consequently of the need for higher therapeutic doses [13].

## 3. Anticancer Agents during Pregnancy

### 3.1. Platinum Compounds

Platinum compounds induce the formation of DNA crosslinks, which interrupt DNA replication and transcription with consequent cellular apoptosis. Furthermore, they form covalent DNA adducts with other subcellular components, such as proteins, lipids, RNA and mitochondrial RNA.

#### 3.1.1. Cisplatin

Cisplatin is the oldest member of platinum compounds, characterized by a low molecular weight and a high binding to plasma or tissue proteins (80–88%). Its half-life is about ten days, so after the administration, cisplatin progressively accumulates in the plasma and shows renal excretion. An in vitro study about the placental transfer of cisplatin showed a negligible transport: only 9% of the maternal drug arrives in the fetal circulation. However, this study should be interpreted with caution, because a short perfusion time of five minutes was used in this in vitro model [83]. The transplacental transfer of cisplatin in pregnant mice changes with the gestational age, increasing during later stages of gestation [84]. Cisplatin has been found in the fetal and neonatal tissues of monkeys and rats [85]. In human umbilical cord blood and amniotic fluid, cisplatin concentrations were found, respectively, 31–65% and 13–42% of that in maternal blood [86]. During pregnancy, cisplatin has been used for the treatment of cervical cancer, ovarian cancer and non-small cell lung cancer (NSCLC) [87]. Regarding the effects on the fetus, a case of a child with severe bilateral perceptive hearing loss was reported after intrauterine exposure to cisplatin during the second and third trimesters of pregnancy [15]. However, most of the literature agrees on the safety of cisplatin administration during the second and third trimesters of pregnancy. A case report regarding the treatment with cisplatin (in association with etoposide) for NSCLC in a pregnant woman during the second and the third trimester described the delivery of a baby in good conditions, without other information about its long-term outcome [14]. Cardonick et al. reported hearing loss in only one that had a genetic predisposition, among seven women treated during the second and third trimester with cisplatin for different malignancies [31]. The use of cisplatin during the second and third trimesters of pregnancy appears to be safe and not related to an increased risk of malformations in children of women treated for ovarian or cervical cancer [88]. Zagouri et al. analyzed 47 pregnant patients treated with cisplatin administered as monotherapy or combined with bleomycin, 5-fluorouracil, paclitaxel, vincristine and bleomycin. All children were healthy, with a median follow-up of 12.5 months. Moreover, in the majority of women, chemotherapy was well tolerated and the median progression-free survival was 48.5 months [89]. Only one baby with cerebral ventriculomegaly and cerebral atrophy is described after intrauterine exposure to cisplatin (with bleomycin and etoposide) for maternal ovarian cancer during the second and third trimesters of pregnancy, but its etiology is not clear [16]. In a systematic review of 43 cases of pregnant women treated with platinum compounds, among 36 patients who received cisplatin, two fetal malformations occurred (microphthalmos and ventriculomegaly), but the role of cisplatin is not clear, because of the short period between malformation time and cisplatin administration [90,91].

#### 3.1.2. Carboplatin

Carboplatin is a platinum compound of relatively low molecular weight (371 g/mol), mainly excreted by the kidneys. Only 24–50% of plasmatic carboplatin is bound to plasma proteins, so its high free fraction and low molecular weight may explain its extensive transplacental passage. Van Calsteren et al. showed carboplatin fetal concentrations of up to 57.5% of the maternal concentration in the baboons’ model and up to 117% in the rats’ model [81,92]. During the pregnancy, carboplatin has been used in cervical and ovarian cancer (in association with bleomycin and etoposide or etoposide) [86], and in the rare NSCLC [93]. Regarding the effects on the fetus, Tabata et al. described normal growth and no evidence of disease in a baby born by a woman with ovarian cancer treated with carboplatin from the 21th week of gestation until the delivery at 33 weeks of gestation [17]. In follow-up periods of 12–18 months, several other studies evidence the absence of any complications in babies born by mother who received carboplatin chemotherapy for epithelial ovarian cancer during the second and third trimesters of pregnancy [18]. Only one case of congenital malformation (gastroschisis) has been described in a fetus who underwent spontaneous miscarriage at the 20th week of pregnancy: the mother was treated with carboplatin after the first trimester for a recurrent CNS malignancy. In conclusion, the latest guidelines seem to be in favor of the use of platinum derivatives after the first trimesters of pregnancy, giving priority to carboplatin, when possible, because of its favorable fetal toxicity profile (such as the minor risk of ototoxicity) [19].

#### 3.1.3. Oxaliplatin

Oxaliplatin is the youngest platinum compound, with a half-life of about ten days, but without accumulation in the plasma; on the contrary, the erythrocytes represent an important deep compartment for oxaliplatin. During pregnancy, oxaliplatin has been used in gastrointestinal cancers, especially in the FOLFOX regimen (which includes calcium folinate, 5-fluorouracil and oxaliplatin) for colorectal carcinoma [94]. To our knowledge, no study has focused on the transplacental transfer of oxaliplatin. There are case reports only about the use of the FOLFOX regimen during the second and third trimesters of pregnancy, without any congenital malformations observed [20]. Furthermore, reports describe the birth of a neonate small for gestational age [21], another affected by hypothyroidism [22] and one case of intrauterine fetal loss at the 33rd week of gestational age [23].

### 3.2. Alkylating Agents

Alkylating agents are chemotherapeutic drugs that determine DNA cross-links and strand breaks binding covalently to DNA via an alkyl group.

#### 3.2.1. Cyclophosphamide

Cyclophosphamide is an anticancer and immunosuppressive drug. Cyclophosphamide is an inactive prodrug, requiring hepatic microsomal enzymatic bioactivation to form 4-hydroxycyclophosphamide (4-OHCP), which has the cytostatic activity. It has a low degree of plasma protein binding, with consequent easy and complete penetration of transplacental membranes. Conversely, 4-OHCP is more strongly bound to plasma proteins, with a lower transplacental passage. In the baboon model, a cyclophosphamide placental transfer has been documented, with comparable fetal and maternal plasma levels within the first 2 h after infusion, whereas the fetal plasma levels of the active metabolite were about 25% of maternal plasma concentrations [81]. In humans, an in-vivo study confirmed in amniotic fluid the 25% of maternal plasma concentration of cyclophosphamide one hour after taking the drug [95]. During pregnancy, cyclophosphamide can be used for the treatment of breast cancer and Hodgkin’s lymphoma. Regarding fetal outcomes, case reports or larger case series provide strong evidence for the teratogenic effects of cyclophosphamide used during the first trimester: absent toes/thumbs, single coronary artery, imperforate anus, umbilical hernia, cleft palate, multiple eye abnormalities, esophageal atresia, later developed thyroid cancer at age 11 years and neuroblastoma at age 14 years [24]. After exposure to multidrug regimens containing cyclophosphamide after the first trimester, a case of rectal atresia and a case of hip subluxation was found. Cardonick et al. analyzed 110 pregnant women treated with multidrug regimens, containing cyclophosphamide and they found 1 intrauterine death (normal fetal autopsy), 1 neonatal death (due to autoimmune disorder), 1 IgA deficiency, 1 pyloric stenosis, 1 holoprosencephaly, 7 IUGR [25]. One of the major concerns is the inability to explore the teratogenic effects of cyclophosphamide from those of other drugs administered as combination therapy [96]. On the contrary, two other studies report no congenital malformation in neonates born after intrauterine exposure to chemotherapy containing cyclophosphamide during the second and third trimesters [97]. Another study on 18 pregnant women exposed to systemic chemotherapy showed a major risk of preterm birth (mean gestational age at birth 35.7 ± 2 weeks) but confirmed the absence of no stillbirths or congenital malformations when cyclophosphamide is used after the first trimester [26]. Finally, a recent study demonstrated that prenatal exposure to cyclophosphamide does not impact fetal brain growth [98].

#### 3.2.2. Ifosfamide

Ifosfamide is an antineoplastic drug chemically related to cyclophosphamide. It has high lipid solubility and a lack of protein binding. Data on transplacental transport of ifosfamide are limited. A recent study on animal models investigated the role of ifosfamide on pregnancies of female rats exposed before mating. Treatment with high doses of ifosfamide caused small placentas, fewer viable fetuses, greater post-implantation losses and more resorbed fetuses. Reduced progesterone and increased prolactin levels also were found [99]. During the pregnancy, ifosfamide may be used in polychemotherapy for the treatment of Ewing sarcoma and soft tissue sarcoma, but limited data on the safety of this drug during pregnancy have been published. An old case report described the development of anhydramnios and complete intrauterine growth following the administration of ifosfamide (in association with vincristine and actinomycin D) during the second and third trimesters of pregnancy, with consequent premature birth and neonatal death. Large areas of ischaemic necrosis of the placenta were found, without kidney malformation in the fetus [27]. Contrariwise, a case study of nine patients treated with the association of doxorubicin and ifosfamide during the second and third trimester of pregnancy showed favorable outcomes for the neonates [28].

#### 3.2.3. Dacarbazine

Dacarbazine is a cell cycle nonspecific antineoplastic alkylating, with minimal plasma protein binding and is metabolized into an active metabolite. A study on the pharmacokinetics of dacarbazine during pregnancy showed that pregnancy decreases the metabolism of the drug, resulting in increased concentrations of dacarbazine and lower exposure to its active metabolites [100]. To our knowledge, there have been no human studies on the placental transfer of dacarbazine in the literature [81]. During the pregnancy, dacarbazine can be used with interferon-alpha for the second or third-line therapies of melanoma and chemotherapy for Hodgkin’s lymphoma. One study analyzed a cohort of 43 pregnant patients treated for Hodgkin’s lymphoma also in the first trimester, demonstrating that chemotherapy with ABVD regimen (adriamycin, bleomycin, vinblastine and dacarbazine) is associated with excellent outcomes for both mothers and children and no clinical malformations were observed; also, the development of newborns was physiological without evidence of cardiac and neurological damage [29]. No congenital malformations have been described also in a cohort of 16 women treated for Hodgkin’s disease in all three trimesters [30]. Instead, Cardonick et al. analyzed 20 women with lymphoma treated with ABVD during the second and third trimesters and found a case of plagiocephaly and one of syndactyly [31].

#### 3.2.4. Busulfan

Busulfan is an alkylating agent, used as a myeloablative-conditioning regimen before stem cell transplantation. An old study found an increased frequency of chromosome aberrations in bone marrow, oocytes, and liver cells of embryos of mice exposed to busulfan [101]. Teratogenic effects of busulfan on rats are also described, such as microencephaly, microphthalmia, microtia, micrognathia, microabdomen, brachydactylia, polydactyly, syndactyly, cleft hand or foot [102]. Furthermore, it has been recognized to induce neural progenitor cell damage in fetal rat brains [32]. Microphthalmia, bilateral corneal opacities, cleft palate, thyroid and parathyroid agenesis, ovarian dysgenesis, microabdomen, pulmonary dysgenesis were reported in a human newborn exposed in utero to 6-mercaptopurine and busulfan. One case of pyloric stenosis and another of unilateral renal agenesis with liver calcifications were reported among two newborns exposed to busulfan during the second trimester [33].

#### 3.2.5. Procarbazine and Mechlorethamine (Chlormethine)

Procarbazine is an alkylating agent with renal and hepatic metabolism and half-time of an hour, used as neoadjuvant or adjuvant chemotherapy for anaplastic astrocytoma and anaplastic oligodendroglioma (in association with lomustine and vincristine). Procarbazine is also adopted in association with mechlorethamine (which belongs to the group of nitrogen mustard alkylating agents) for the treatment of Hodgkin’s disease in MOPP regimen (Mechlorethamine, Vincristine, Prednisone, Procarbazine) [103]. In rat models, procarbazine seems to affect intrauterine fetal development, without any teratological effects [104]. Another study showed the occurrence of clefts in the palate area after the administration of procarbazine to rats during the pregnancy [105]. In humans, Aviles et al. described the absence of any congenital malformations detected in newborns from women treated with MOPP regimen for Hodgkin’s disease during all the trimesters of pregnancy [97]. A case of hydrocephalus and perinatal death occurred after prenatal exposure to MOPP regimen during the first trimester; moreover, a case of syndactyly occurred after prenatal exposure to MOPP regimen during the second trimester [34]. Blumenthal et al. reported the case of a healthy infant after prenatal exposure to the procarbazine–lomustine–vincristine regimen for the treatment of maternal malignant glioma [35].

#### 3.2.6. Chlorambucil

Chlorambucil is a direct-acting cytotoxic agent that does not require any metabolic activation and is used for the treatment of chronic lymphocytic leukemia and Hodgkin’s lymphoma. In rat models, teratogenic effects are associated with prenatal exposure to chlorambucil, such as alterations of prosencephalon or limb, tail, renal, cranial and axial skeleton abnormalities [106]. In humans, the use of chlorambucil during pregnancy has been described only in two reports, that reported normal infants without any abnormality [36].

### 3.3. Antimetabolites

Antimetabolites are anticancer drugs that act in the S phase of the cell cycle as a false substrate during DNA and RNA synthesis; this results in the formation of truncated cellular proteins.

#### 3.3.1. Cytarabine

Cytarabine (Cytosine arabinoside, Ara-C) is a prodrug with low molecular weight, that requires the conversion into an active metabolite (cytarabine-5′-triphosphate) which inhibits the DNA polymerase. Moreover, it is incorporated into DNA and RNA, impairing their synthesis and function. This anticancer drug is adopted for the treatment of acute lymphocytic and non-lymphocytic leukemia, and chronic myelogenous leukemia. Transplacental transport of cytarabine is documented in mice. Cytarabine may induce teratogenic effects in mice, rats and chicks, including skeletal defects, cleft palate, cerebellar hypoplasia, microcytic renal changes and retinal dysplasia [107]. In humans, teratogenic effects of intrauterine exposure to cytarabine have been documented over all three trimesters in various case reports, such as skeletal malformations of the face, skull and limbs, ear defects, atrial septal defects, cardiomyopathy [37]. Furthermore, recurrence of intrauterine growth restrictions and intrauterine death have been described [38].

#### 3.3.2. Methotrexate

Methotrexate is an antineoplastic drug that inhibits the enzyme dihydrofolic acid reductase and consequently the synthesis of purine nucleotides and thymidylate which allow DNA replication and repair. Methotrexate has an elevated molecular weight and it’s bound to the plasma proteins for 50% [108]. It’s used for the treatment of neoplastic diseases (leukemia, breast cancer, epidermoid cancers of the head and neck, lung cancers, non-Hodgkin lymphoma, osteosarcoma), autoimmune and dermatologic diseases, and ectopic pregnancies [109]. Teratogenic effects were documented after high doses of methotrexate, but more recent studies have demonstrated that spontaneous miscarriage and birth defects may occur even after prenatal exposure to low-dose during the first trimester of pregnancy [39]. Skeletal abnormalities (cranial dysostosis with delayed ossification, hypertelorism, wide nasal bridge, micrognathia, microcephaly, abnormalities of the limbs), cardiac defects (Tetralogy of Fallot, pulmonary valve atresia), ear anomalies, ambiguous genitalia are described after early methotrexate exposure during pregnancy [40]. Fetal methotrexate syndrome was an embryopathy with craniofacial, cardiac, pulmonary, gastrointestinal, genitourinary and musculoskeletal anomalies. A minor incidence of congenital malformations due to the administration of methotrexate during the last two trimesters of pregnancy was observed, consequently, its use is contraindicated during pregnancy [41,42].

#### 3.3.3. Gemcitabine

Gemcitabine is an analog of cytarabine, from which it differs structurally due to its fluorine substituents on position 29 of the furanose ring. Like Ara-C, gemcitabine is a prodrug that requires cellular uptake and intracellular phosphorylation [110]. Transplacental transfer of gemcitabine is documented in rat models, with a major risk of toxicity when high doses are administered [111]. Gemcitabine may be used for the treatment of advanced biliary tract cancers (with platinum compounds in the GEMOX regimen), pulmonary adenocarcinoma or non-small-cell lung carcinoma, and pancreatic adenocarcinoma. It has been administered during the first trimester of pregnancy for a pulmonary adenocarcinoma, without any negative effect on the child. No evidence of congenital malformations is reported after the administration of gemcitabine during the last two trimesters of pregnancy, but cases of IUGR are described [43].

#### 3.3.4. 5-Fluoruracil (5-FU)

5-FU is an anticancer drug with low molecular weight and negligible protein binding. As yet, there are no data about its transplacental transfer in humans. On the contrary, one study about its transplacental transfer in a rat model revealed that a significant amount of 5-FU crossed the placenta, with fetal exposure that increased in a dose-dependent manner. 5-FU was poorly eliminated in the rat fetus, thus fetal toxicity results lower than maternal toxicity at dosage levels [112]. 5-FU can be used for chemotherapy of breast cancer and colorectal carcinoma in FOLFOX or FOLFIRI regimens (that include 5-FU, leucovorin and irinotecan). Based on the literature, prenatal exposure to 5-FU seems to be associated with a higher risk of fetal congenital malformations in the first trimester than in the last two trimesters. In addition, exposure to 5-fluorouracil in the first trimester increases the rate of spontaneous abortion (incidence of 25% versus 13% of the general population) [45]. The exposure during the first trimester of pregnancy is associated with major malformations, such as microcephaly, ventriculomegaly, colpocephaly, hypertelorism, flat nasal bridge, skeletal deformities of the hand and feet (including syndactyly and hypoplasia of the digits), bicuspid aortic valve [46].

#### 3.3.5. Capecitabine

Capecitabine is a prodrug metabolized to 5-FU. Capecitabine has low molecular weight and a moderate plasma protein-binding (it is bound to albumin for about the 35%). During pregnancy, it can be used for the treatment of solid tumors such as colorectal and breast cancers. Literature about the effect of fetal exposure to capecitabine is scarce. No congenital malformations were described in a baby born from a woman treated with capecitabine and oxaliplatin during the first trimester of pregnancy for colorectal cancer, but once the pregnancy was discovered treatment was stopped. The child was reported to be healthy at two years of age. A pregnant patient affected by high-grade neuroendocrine carcinoma was treated with capecitabine during the first trimester of pregnancy, without any consequence for the newborn [44].

##### 3.3.6. 6-Mercaptopurine

6-Mercaptopurine is an anti-metabolite drug, with low molecular weight and poor binding with plasma proteins. Transplacental transfer of 6-MP has not been studied in humans. 6-MP is used for the treatment of acute lymphocytic leukemia. No congenital malformations were described in fifty women treated with 6-MP-containing regimens during all three trimesters of pregnancy. However, four cases of IUGR, one of intrauterine death and two cases of neonatal death were reported. To our knowledge, there were no major congenital malformations following the exposure to 6-mercaptopurine after the first trimester [30].

### 3.4. Antitumor Antibiotics

This is a group of anticancer drugs that work in all phases of the cell cycle binding DNA, preventing RNA synthesis, inhibiting the enzyme topoisomerase and generating highly reactive free radicals that damage intercellular molecules.

#### 3.4.1. Anthracyclines: Daunorubicin, Doxorubicin, Epirubicin, Idarubicin

Daunorubicin is an anthracycline with high molecular weight. The presence of daunorubicin in the organ tissue of a dead fetus exposed in utero was reported, but no other data about its transplacental transfer are available [113]. Daunorubicin is used for the treatment of acute lymphocytic and non-lymphocytic leukemia. When administered during pregnancy, it is associated with the development of congenital malformations during both the first trimester and the last two trimesters. Ocular and cardiac defects, skeletal malformations of the distal limbs and cranium, and premature closure of cranial sutures were reported after early exposure. Syndactyly and rectal atresia were reported after later exposure [47].

Doxorubicin (Adriamycin) is an anthracycline with high molecular weight and high binding with plasma proteins (about 70–75%). Animal models show an average maternofetal transfer rate from 4% to 7.5% [81]. In human in-vivo studies, doxorubicin is detectable in fetal organs at the delivery, but not in cord blood, amniotic fluid or placenta [114]. PEGylated liposomal doxorubicin doesn’t seem to be able to cross the placental barrier, as opposed to the non-PEGylated liposomal doxorubicin [115]. Doxorubicin is indicated for the treatment of hematological cancers (acute lymphoblastic leukemia, acute myeloblastic leukemia, multiple myeloma, Hodgkin lymphoma), solid tumors (cancers of breast, ovary, stomach, thyroid, Wilms tumor, neuroblastoma, soft tissue and bone sarcomas, transitional cell bladder cancer and bronchogenic carcinoma). Both mouse model studies and in vitro studies on human placental tissue, explants and trophoblast cells demonstrated the toxicity of doxorubicin on cells and tissue viability [116]. In mouse models, the administration of doxorubicin during pregnancy led to offspring’s brain development and behavior impairments [117,118,119]. According to several case reports, in humans, the use of doxorubicin appears relatively safe during pregnancy [58,120]. Brito et al. demonstrated that exercise performed by mothers protects the neonatal heart against doxorubicin-induced toxicity, inducing the modulation of antioxidant enzyme and the expression of SIRT6 protein, which inhibits cardiomyocyte hypertrophy, formation of atheromatous plaque and infiltration of inflammatory cells, ameliorating the cardiac remodeling and preventing the development of cardiovascular diseases [54]. As shown by Gziri et al., no significant differences in conventional cardiac measurements, TDI velocities, and strain measurements were found between patients exposed to fetal anthracyclines and controls [48]. Furthermore, Aviles et al. evaluated 81 children whose mothers were treated with anthracyclines during pregnancy (70 with doxorubicin) to detect cardiac toxicity and they did not find any clinical or echocardiogram evidence of late cardiac toxicity [49]. No congenital malformations, cardiotoxicity, growth and development impairment were reported in two studies, which analyzed the outcome of pregnant women with hematological malignancies treated with doxorubicin during pregnancy [121]. Similarly, no congenital malformations were detected in hundred cases of women treated with doxorubicin and cyclophosphamide during the second and third trimesters of pregnancy. Congenital malformations observed after the intrauterine exposure to doxorubicin occurred during the first trimester and consisted of skeletal malformations of digits or cranial bones, imperforate anus and rectovaginal fistula [122]. Epirubicin is an anthracycline with high molecular weight and it binds to the plasma proteins (about 77%). Epirubicin is indicated for the treatment of breast cancer. Placental transport of epirubicin is unknown in humans, but it has been documented in animal models [81]. A case of intrauterine death after exposure to epirubicin used for breast cancer has been reported. Fetal epirubicin exposure during the first trimester is associated with a rate of major malformations of about 20%, with the occurrence of micrognathia, syndactyly and other fingers and metatarsal abnormalities [50]. Moreover, major malformations, such as polycystic kidney, clubfoot and rectal atresia were reported after fetal exposure also after the first trimester [51]. Parodi et al. describe the case of a newborn who developed transient ventricular hypokinesia after in utero exposure to four epirubicin cycles for pregnancy-associated breast cancer. Epirubicin was administered from the 25th week of gestational age and an elective cesarean delivery was planned 3 weeks after the last cycle of chemotherapy at 36 + 6 weeks of gestational age. Echocardiography at four days of life showed bilateral ventricular hypokinesia, which completely disappeared at the ultrasound performed at 1 and 6 months of life [123]. Framarino-dei-Malatesta et al. reported the death of one twin (who was small for gestational age and in oligohydramnios) and a transient cardiotoxicity (with high levels of troponin and transient left ventricular septal hypokinesia) in the surviving fetus after the administration of two cycles of epirubicin for maternal breast cancer [122].

Idarubicin is an anthracycline as daunorubicin, with high molecular weight, great bound to the plasma proteins (for 90–94%) and elevated lipophilicity, which allows a higher rate of cellular uptake than other anthracyclines. It has been hypothesized that its high liposolubility and long half-life may facilitate the transplacental transport. However, after the delivery, undetectable levels of the drug (administered two weeks before) have been found in the maternal serum and umbilical cord blood samples. Idarubicin is indicated for the treatment of acute myeloid leukemia. Its administration during the first trimester of pregnancy doesn’t seem to be associated with any congenital malformations [52]. However, fingers and limbs malformations and micrognathia were reported after exposure to idarubicin from the 21st week of pregnancy, making unclear its associations to the malformations. Fetal and neonatal dilatative cardiomyopathy after the exposure to idarubicin during the last two trimesters has been documented in several studies [53].

#### 3.4.2. Other: Actinomycin-D, Bleomycin

Actinomycin D is a cytotoxic antibiotic with high molecular weight, produced by Streptomyces parvulus. It binds DNA and inhibits RNA synthesis. Actinomycin D is reported to be embryotoxic and teratogenic in mice, chicks and hamsters [124]. There is poor literature about the use of actinomycin-D during human pregnancies. However, a clubfoot occurred in a newborn after exposure to actinomycin-D during the second and third trimesters [125].

Bleomycin is an antitumor antibiotic isolated from the bacterium *Streptomyces verticillus*, with high molecular weight and very low plasma protein binding. Bleomycin generates free radicals which cause DNA strand breaks and inhibits the ligase enzyme that repairs DNA strand breaks. Bleomycin is indicated for the treatment of Hodgkin’s lymphoma and ovarian cancer (in BEP regimens). No congenital malformations were detected in newborns from women with lymphoma treated with bleomycin during all three trimesters of pregnancy [54]. However, some studies describe the occurrence of malformations after the exposure to bleomycin during the first trimester (floating thumb malformation) and during the second and third trimester (plagiocephaly and syndactyly) [31].

### 3.5. Topoisomerase Inhibitor

Topoisomerase inhibitors interfere with the action of topoisomerases, which are enzymes involved in cellular division and DNA replication.

#### 3.5.1. Topoisomerase I Inhibitors: Irinotecan

Irinotecan is a topoisomerase I inhibitor, which binds to the DNA-topoisomerase I complex, preventing the resealing of the DNA. It may be used alone or in combination with additional chemotherapy or targeted therapy. Irinotecan is converted to SN38, its active metabolite. Irinotecan and SN38 have molecular weights which allow transplacental passage, but currently, no data have been published about the transplacental passage of irinotecan in animal or human models. Irinotecan adopted during pregnancy for the treatment of colorectal cancer and ovarian cancer after the first trimester doesn’t seem to be associated with congenital malformations. Taylor et al. reported the birth of a healthy child from a 34-years-old woman affected by ovarian Krukenberg tumor, who underwent chemotherapy with 5-fluorouracil, folinic acid and irinotecan from the second trimester until the 36th week of pregnancy [55]. Moreover, the absence of abnormalities was found in an infant born from a 33-years-old mother affected by metastatic colon adenocarcinoma, who received irinotecan and 5-Fluorouracil from the 23th to the 28th week of gestational age [56].

#### 3.5.2. Topoisomerase II Inhibitors: Etoposide

Etoposide binds to topoisomerase II and DNA, preventing the resealing of DNA breaks with consequent inhibition of DNA replication and cell death. No data are available about its transplacental passage neither in animals or humans, but in their study, Yamauchi et al. demonstrated that the injection of etoposide to pregnant mice induced placental apoptosis and severe intrauterine growth restriction [126]. Etoposide has been used in several cases of ovarian cancer that occurred during pregnancy, in association with bleomycin and cisplatin after the first trimester [127]. No case of congenital malformation occurred, except for the case of a child born prematurely at the 28th week of pregnancy from a mother affected by an endodermal sinus tumor. At 16 months after delivery, the infant developed significant ventriculomegaly with cerebral atrophy, of which etiology remains unclear [57]. Etoposide has been adopted also for the management of hemophagocytic lymphohistiocytosis during pregnancy, a rare and severe syndrome that requires the adoption of etoposide for the cases refractory to corticosteroids [128]. However, further studies are required to identify its appropriate dosage and the correlation between dose and side effects (such as myelosuppression) on the fetus [129].

### 3.6. Antimitotic Agents

Antimitotic agents stop mitosis in the phase M of the cell cycle; the polymerization of microtubules is prevented by plant alkaloids, whereas their depolymerization is inhibited by taxanes.

#### 3.6.1. Plant Alkaloids: Vincristine and Vinblastine

Vincristine and vinblastine are alkaloids derived from the periwinkle plant, metabolized in the liver and excreted mainly by the biliary and intestinal tracts. They show a high protein bound and they are substrates for PGP and MRP transporters [130]. Transplacental transfer of vinblastine and vincristine in humans has not been studied yet, but the fetal concentration was found, respectively, 18.5% and 14% of the maternal concentration in baboons and mice [81]. Vinblastine and vincristine are crucial for the treatment of acute lymphoblastic leukemia (ALL), Hodgkin’s lymphoma and other lymphomas and they are used mostly in polychemotherapy regimens [130]. There are many case reports regarding healthy children following therapy with vincristine alone or combined with other drugs for the treatment of hematological malignancies during all three trimesters. Exposure to vincristine during the first trimester has been also associated also with the development of atrial septum defect, bilateral radius and fifth digit absence, hydrocephalus, renal and cardiac abnormalities and intrauterine death, 46 karyotypes with gaps and a ring chromosome [58]. There are various case reports concerning the use of vinblastine even during the first trimester, mostly in combination with other cytostatic drugs. In most cases, the course of pregnancy was normal and single-agent vinblastine used as monotherapy appears to be preferred to polychemotherapy [131]. Mulvihill et al. report children with malformations that occurred in the first trimester after the exposure to vinblastine alone: a child with hydrocephalus, a spontaneous miscarriage and another with cleft palate [59]. There is also a report of an infant delivered at 37 weeks from a mother treated with vincristine and vinblastine, weighing 1900 g with an atrial septum who eventually died because of respiratory distress syndrome [132].

#### 3.6.2. Taxanes: Paclitaxel and Docetaxel

Paclitaxel and docetaxel are semisynthetic taxanes, which bind to tubulin, leading to microtubule stabilization, mitotic arrest and cell death. They exhibit high molecular weight, high lipo-solubility and are highly protein-bound. Consequently, their distribution to the tissues is fast, with low plasma levels and slow clearance. Taxanes are widely metabolized in the liver by the cytochrome P-450 enzymes (CYP2C8 for paclitaxel and CYP3A4/5 for docetaxel). Although the fetal liver does not express cytochrome P-450 enzymes yet, the maternal liver increases its production during the third trimester, inducing fetal tolerance to these agents. Furthermore, they are substrates of PGP and MRP, which limit their transfer and storage into the fetus. In the baboon model docetaxel was not detected in fetal blood samples and paclitaxel was found in very little plasma concentration (1.6 ± 0.8) [81]. The therapeutic use of taxanes during pregnancy has been widely described in pregnant women with breast, cervical and ovarian cancers, with tolerable profiles especially during the second and third trimesters [133,134]. In their systematic review, Mir et al. collected updated data about the use of taxanes in 40 pregnant patients, in almost all cases administered with other cytotoxic agents (anthracyclines, cyclophosphamide and platinum compounds) [135]. They found only one case of pyloric stenosis possibly related to taxanes in a neonate born from a woman who had received polychemotherapy with doxorubicin, cyclophosphamide, paclitaxel and docetaxel [60]. Taxanes show a toxicity profile more favorable than anthracyclines or etoposide-based regimens, but further clinical and pharmacokinetic examinations are required for defining their optimal dosing regimen in pregnant patients [136]. Cardonick et al. collected meconium samples from 23 newborns whose mothers underwent chemotherapy for cancer and analyzed them for metabolites of chemotherapeutics through liquid chromatography-high resolution mass spectrometry. Paclitaxel and its metabolites (3′-p-hydroxypaclitaxel and 6α-hydroxypaclitaxel) were found in 8 samples, with variability in meconium levels between samples, based on timing, dosing and individual characteristics [137].

### 3.7. Targeted Agents

Targeted agents currently adopted for the treatment of several and different types of cancer vary from monoclonal antibodies (large molecules that require active transporter for the transplacental passage) to tyrosine kinase (TK) inhibitors (small molecules similar to chemotherapy that may cross the placenta throughout the pregnancy period). They could also produce very specific pregnancy-related adverse events secondary to their target effect against tumor-specific molecular aberrations which still may play a role in fetal development [138]. Burotto et al. described the first case of a pregnant woman with metastatic melanoma treated with nivolumab and ipilimumab. The patient underwent a cesarean section giving birth to a healthy newborn, with no clinical signs of melanoma, achieving disease stability in the PET-CT performed 6 months after delivery [139]. Nevertheless, for these new agents, such as ipilimumab, nivolumab, pembrolizumab, dabrafenib, trametinib, vemurafenib, erlotinib and gefitinib, not much data are currently available and more evidence is required for considering their use during pregnancy [61].

#### 3.7.1. Rituximab

Rituximab is a chimeric antiCD20 monoclonal antibody used for the treatment of B-lymphoproliferative diseases (relapsed or refractory, low-grade or follicular, CD20+, B-cell non-Hodgkin lymphoma, diffuse large B-cell non-Hodgkin lymphoma, CD20+ chronic lymphocytic leukemia, as a single agent or in combination with chemotherapy) and autoimmune disorders (systemic lupus erythematosus, idiopathic thrombocytopenic purpura, thrombotic thrombocytopenia, multiple sclerosis). In monkey models, Rituximab crosses the placenta, without teratogenic effects. Transient depletion of peripheral B-cell is observed, but it resolves usually within 6 months after the exposure to Rituximab [140]. Several reports described generally reassuring neonatal outcomes after maternal exposure to rituximab during pregnancy, often in conjunction with combination chemotherapy, for the treatment of malignancies or severe nonmalignant hematologic abnormalities [141]. Chakravarty et al. analyzed 231 women exposed to Rituximab during pregnancy for the treatment of lymphoma or autoimmune diseases. Only the data of 153 of them were available and they had to be analyzed with caution, considering the concomitant use of other potentially teratogenic drugs and severe underlying illnesses. They found only two congenital malformations: a cardiac malformation (ventral sept effect, with patent foramen oval and ductus arteriosus) and a clubfoot in one twin. Moreover, eleven cases of hematologic abnormalities (peripheral B-cell depletion, neutropenia, lymphopenia, thrombocytopenia and anemia) were reported, almost all transient, that recovered spontaneously within weeks or months, except for the case of an infant born from a mother with idiopathic thrombocytopenic purpura, who had a cerebral hemorrhage. Finally, they described four cases of neonatal infections: one fever of suspected viral origin, acute hepatitis in a mother with cytomegalovirus infection, bronchiolitis and acute chorioamnionitis [142]. Several studies have demonstrated that Rituximab passes the human placenta transiently, inhibiting neonatal B-Lymphocyte development; however, after the first 6–10 months, B-lymphocytes levels return to normal, without causing functional deficits or inadequate vaccination IgG titers in the infants [62,143].

#### 3.7.2. Trastuzumab

Trastuzumab is a humanized IgG1 monoclonal antibody used in epidermal growth factor receptor 2 (HER-2) positive breast cancer; its major side-effect is cardiotoxicity (reversible maternal heart failure, which resolves after its discontinuation). It passes the placenta and has been found in significant concentrations in fetal plasma of baboon models (85% at 2 h after trastuzumab infusion) [81]. Currently, in humans the use of trastuzumab during pregnancy is not recommended, because it has been associated with oligo/anhydramnios and consequent fetal renal insufficiency, which potentially related to HER-2 and vascular endothelial growth factor (VEGF) inhibition in fetal nephrogenic cells, with decreased renal blood flow [144]. In their systematic review, Andrikopoulou et al. synthesized currently available data about the effect of trastuzumab on fetal and maternal outcomes. Oligo/anhydramnios was found as the most frequent side effect (in 58.1% of women) especially if administered after the first trimester, as reported in National Toxicology Program monograph [108]. The transplacental passage of trastuzumab seems to be minimal during the first trimester, when the placental expression of FC receptor (to which the placental active transporter of trastuzumab binds) is minimal [63].

#### 3.7.3. Lapatinib

Lapatinib is a dual inhibitor of the EGFR and HER-2, used as a single agent or in combination with trastuzumab for the treatment of patients with HER2-positive metastatic breast cancer. Kelly et al. reported the case of a woman with metastatic HER2 mutated breast cancer disease, who became accidentally pregnant during the treatment with lapatinib. Lapatinib was interrupted at the 14th week of gestation, but no pregnancy complications or fetal malformations were observed [145].

#### 3.7.4. Bevacizumab

Bevacizumab is a recombinant humanized monoclonal immunoglobulin antibody with two antigen-binding domains that block all active forms of VEGF-A, inhibiting endothelial cell proliferation and angiogenesis in a variety of solid tumors [146]. It shows an inhibitory effect on pregnancy development in rat models and teratogenic effects in rabbits [64]. Furthermore, bevacizumab is expected to induce IUGR, due to the inhibition of VEGF which is crucial for vascular permeability. Consequently, its use during pregnancy is controversial because of the potential systemic side effects on the mother and fetal harm, such as preeclampsia and spontaneous miscarriage, respectively. In the literature, there are only available studies about its intravitreal injection in women with diabetic retinopathy and choroidal neovascularization and cases of preeclampsia and miscarriages are reported [65]. Due to the lack of extensive data, bevacizumab should be used with extreme caution during pregnancy, until more data become available in humans [66].

#### 3.7.5. Imatinib, Nilotinib and Dasatinib

Imatinib is a tyrosine-kinase (TK) inhibitor, a molecularly targeted therapy, which has become a milestone in the treatment of chronic myeloid leukemia (CML). TKs inhibitors are active against several TKs, which are signaling molecules that regulate cellular proliferation, differentiation, survival, function and motility. Various tumors overexpress TKs, leading to uncontrolled mitogenic signals to the neoplastic cells. Animal studies showed its teratogenicity in rats (causing defects such as exencephaly, encephaloceles, and deformities of the skull bones) and congenital malformations have been correlated with its use during human organogenesis [67]. Pye et al. analyzed 12 pregnancies of women treated with imatinib from the first trimester for CML; three of them underwent elective termination. They reported 1 stillbirth and 8 live births, all with congenital malformations (such as craniosynostosis, exomphalos, renal agenesis, skeletal abnormalities, hypoplastic lungs, pyloric stenosis, hypospadias, cleft palate, polydactyly, hydrocephalus, cerebellar hypoplasia, cardiac defects, meningocele) [68]. Thus, current recommendations discourage the adoption of imatinib and other second-generation TKs inhibitors (such as dasatinib nilotinib) during the first trimester. After the 16th week of pregnancy, their use may be considered in selected cases. In fact, several studies have described its effectiveness and safety after the first trimester: no congenital malformations or side effects were found [69,147]. Dasatinib and nilotinib have similar safety profiles during pregnancy as imatinib, however, they should be limited during pregnancy before more data will be available [148].

#### 3.7.6. Gefitinib and Erlotinib

Gefitinib and erlotinib are epidermal growth factor receptor (EGFR)-TKs inhibitors, recommended as the first-line therapy for EGFR-mutated lung cancer. Erlotinib is also approved for the treatment of advanced pancreatic cancer. Gefitinib has been used during pregnancy only in two cases in the third trimester, without any fetal or maternal side effects [70]. Erlotinib was adopted without sequelae for the duration of the pregnancy in only one case of a female patient with stage IV lung adenocarcinoma, with mediastinal, bone and cerebral metastasis and EGFR mutation. At the time of observation of the study, the patient was undergoing the 11th month of treatment and the baby was 4 months old and in good health [149].

#### 3.7.7. Vemurafenib

Vemurafenib is a BRAF-inhibitor active against BRAF V600E mutation, which is expressed in approximately 50% of malignant melanoma [150]. In animal studies (rats and rabbits), vemurafenib crosses the placenta without any teratogenic effects. In the literature are reported some cases of women treated with vemurafenib during pregnancy after the first trimester for metastatic melanoma, without any maternal and fetal side effects [151]. Maleka et al. describe the case of one pregnant patient with metastatic melanoma who responded immediately to vemurafenib (with the shrinkage of tumor and normalization of transaminases). The patient had a 3-month progression-free survival and give birth to a healthy newborn on the 30th week of gestation, 5 weeks after the initiation of vemurafenib. A restriction of growth was observed at the 24th week of gestation, before the initiation of the vemurafenib therapy, as a result of malnutrition and catabolic status associated with the maternal illness. Maybe the toxic effect of vemurafenib treatment was a contributing factor to the inhibition of growth [152]. Nevertheless, Vemurafenib may cause toxic epidermal necrolysis (TEN), because it contains a sulfonamide group and cross-reactivity to sulfonamide compounds has been reported in allergic patients. Thus, sulfonamide drugs should be avoided in patients with serious hypersensitivity reactions to vemurafenib and vice versa [153]. De Haan et al. reported the case of a pregnant woman treated with vemurafenib for metastatic melanoma from the 22nd week of pregnancy, who developed a mild skin rash after 12 days after commencing vemurafenib. In the next 9 days the rash progressed to TEN and the woman was admitted to the intensive care unit intubated and sedated. At 26 weeks of gestational age, she spontaneously delivered two preterm baby boys that were healthy and appropriate for gestational age. 53 days after the delivery, the mother finally died of intracranial hemorrhage due to metastases, whereas both children were healthy and developmentally normal at 15 months of age [154].

## 4. Conclusions

PAC has become a clinical multidisciplinary challenge for gynecologists, hematologists or oncologists and neonatologists, that manage women with a neoplastic diagnosis during pregnancy. Chemotherapy can be offered to women with cancer during pregnancy, although it is not without risk to the fetus. Despite the lack of guidelines about the management of PAC, several studies have described the use and the potential fetal and neonatal adverse events of antineoplastic drugs during pregnancy.

## Figures and Tables

**Table 1:** Pregnancy and fetal outcomes after human intrauterine exposure to antineoplastic agents.

Antineoplastic Agents	Maternal Tumor	Pregnancy and Fetal Outcomes
***Platinum compounds***		
**Cisplatin**	Cervical cancer, ovarian cancer, non-small cell lung cancer (NSCLC)	No congenital malformations (T2–T3) [14] Severe bilateral perceptive hearing loss (T2–T3) [15] Cerebral ventriculomegaly and cerebral atrophy (T2–T3) [16]
**Carboplatin**	Cervical cancer, ovarian cancer, NSCLC	No congenital malformations (T2–T3) [17,18] Spontaneous miscarriage of a fetus with gastroschisis (T2–T3) [19]
**Oxaliplatin**	Colorectal carcinoma	No congenital malformations (T2–T3) [20] Small for gestational age at birth (T2–T3) [21] Hypothyroidism (T2–T3) [22] Spontaneous miscarriage at 33 weeks of gestational age (T2–T3) [23]
***Alkylating agents***		
**Cyclophosphamide**	Breast cancer, Hodgkin’s lymphoma	Teratogenic effects and multiple congenital anomalies (T1) [24,25] No congenital malformations (T2–T3) [20] Preterm birth (T2,T3) [26]
**Ifosfamide**	Ewing sarcoma, soft tissue sarcoma	Anhydramnios and intrauterine growth arrest (T2–T3) [27] No congenital malformations (T2–T3) [28]
**Dacarbazine**	Melanoma, Hodgkin’s lymphoma	No congenital malformations (T1–T2–T3) [29,30] Plagiocephaly, syndactyly (T2–T3) [31]
**Busulfan**	Chronic myeloid leukemia, myeloablative-conditioning	Teratogenic effects and multiple congenital malformations (T1–T2–T3) [32] Renal agenesis and liver calcifications (T2) [33]
**Procarbazine** **Mechlorethamine**	Anaplastic astrocytoma, oligodendroglioma, Hodgkin’s disease	Hydrocephaly and perinatal death (T1) [34] Syndactyly (T2) [34] No congenital malformations [35]
**Chlorambucil**	Chronic lymphocytic leukemia, Hodgkin’s lymphoma	No congenital malformations [36]
***Antimetabolites***		
**Cytarabine**	Acute lymphocytic and non-lymphocytic leukemia, chronic myelogenous leukemia	Teratogenic effects (T1–T2–T3) [37] Intrauterine growth retardation (IUGR), intrauterine death [38]
**Methotrexate**	Leukemia, breast cancer, lung cancers, non-Hodgkin lymphoma, osteosarcoma, autoimmune diseases, dermatologic conditions, ectopic pregnancies, pregnancy termination	Spontaneous miscarriage and birth defects (T1) [39] Craniofacial, cardiac, pulmonary, gastrointestinal, genitourinary and musculoskeletal anomalies (T1–T2–T3) [40,41,42]
**Gemcitabine**	Biliary tract cancers, pulmonary adenocarcinoma, non-small-cell lung carcinoma (NSCLC), pancreatic adenocarcinoma	Healthy newborn (T1–T2) [43] IUGR (T2–T3) [43]
**Capecitabine**	Colorectal carcinoma, breast cancer	Healthy children (T1) [44]
**5-Fluoruracil**	Colorectal carcinoma, breast cancer	Spontaneous abortion (T1) [45] Multiple congenital malformations (T1) [46]
**6-Mercaptopurine**	Acute lymphocytic leukemia	No congenital malformations (T1–T2–T3) [30] IUGR, intrauterine and neonatal death (T1–T2–T3) [30]
***Antitumor antibiotics***		
**Daunorubicin**	Acute lymphocytic and myeloid leukemia	Teratogenic effects (T1–T2–T3) [47]
**Doxorubicin**	Hematological cancers, various solid tumors	Skeletal malformations, imperforate anus and rectovaginal fistula (T1) [48,49] No congenital malformations, no cardiotoxicity (T2, T3) [49]
**Epirubicin**	Breast cancer	Intrauterine death, micrognathia, syndactyly, other fingers/metatarsal abnormalities (T1) [50] Polycystic kidney, clubfoot and rectal atresia (T2–T3) [51]
**Idarubicin**	Acute myeloid leukemia	No congenital malformations (T1) [52] Dilated cardiomyopathy, fingers and limbs malformations and micrognathia (T2–T3) [53]
**Bleomycin**	Hodgkin’s lymphoma, ovarian cancer	No congenital malformations (T1–T2–T3) [54] Floating thumb malformation (T1) [31] Plagiocephaly and syndactyly (T2–T3) [31]
***Topoisomerase inhibitor***		
**Irinotecan**	Colorectal cancer, ovarian cancer	No congenital malformations (T2–T3) [55,56]
**Etoposide**	Ovarian cancer, hemophagocytic lymphohistiocytosis	No congenital malformations except one case of ventriculomegaly with cerebral atrophy (T2–T3) [57]
***Antimitotic agents***		
**Vincristine**	Acute lymphoblastic leukemia, Hodgkin’s lymphoma, other lymphomas	Atrial septum defect, bilateral radius and fifth digit absence, hydrocephalus, renal and cardiac abnormalities (T1) [58]
**Vinblastine**	Acute lymphoblastic leukemia, Hodgkin’s lymphoma, other lymphomas	Hydrocephalus, spontaneous miscarriage, cleft palate (T1) [59]
**Paclitaxel, Docetaxel**	Breast cancer, cervical cancer, ovarian cancer	Healthy children (T1–T2–T3) [60] Pyloric stenosis [60]
***Targeted agents***		
**Rituximab**	Cell B-lymphoproliferative diseases, autoimmune disorders	Cardiac malformation, clubfoot, transient hematologic abnormalities (peripheral B-cell depletion, neutropenia, lymphopenia, thrombocytopenia and anemia), neonatal infections [61]
**Trastuzumab**	HER-2 positive breast cancer	Oligo/anhydramnios with consequent fetal renal insufficiency [62]
**Bevacizumab**	Various solid tumors	Possible IUGR, miscarriage [63]
**Imatinib, Nilotinib, Dasatinib**	Chronic myeloid leukemia	Teratogenic effects (T1) [64] No congenital malformations (T2–T3) [65,66,67]
**Gefitinib, Erlotinib**	EGFR-mutated lung cancer	No congenital malformations (T3) [68,69]
**Vemurafenib**	Melanoma	Healthy children (T2–T3) [70]

T1: first trimester; T2: second trimester; T3: third trimester; IUGR: Intrauterine growth restriction.

**Table 2:** Relatively safe and absolutely contraindicated antineoplastic agents.

Antineoplastic Agents	Relatively Safe (After 1st Trimester)	Absolutely Controindicated
***Platinum compounds***	Cisplatin, Carboplatin, Oxaliplatin	-
***Alkylating agents***	Cyclophosphamide, Ifosphamide, Dacarbazine, Procarbazine, Chlorambucile	Busulphan
***Antimetabolites***	Gemcitabine, 5-Fluoruracile, Capecitabine, 6-Mercaptopurine	Methotrexate, Cytarabine
***Antitumor antibiotics***	Doxorubicine, Epirubicin, Bleomycin, Actinomycin-D	Daunorubicine, Idarubicin
***Topoisomerase inhibitors***	Irinotecan, Etoposide	-
***Antimitotic agents***	Vincristine, Vinblastine, Docetaxel, Paclitaxel	-
***Targeted agents***	Rituximab, Lapatinib, Imatinib, Nilotinib, Dasatinib, Gefitinib, Erlotinib, Vemurafenib	Trastuzumab, Bevacizumab
